# The role of microRNAs in brain metastasis

**DOI:** 10.1007/s11060-023-04541-x

**Published:** 2024-01-09

**Authors:** Kadie Hudson, Mark Willy Mondia, Ying Zhang, Shekhar Saha, Myron K. Gibert, Collin Dube, Yunan Sun, Pawel Marcinkiewicz, Camilo Fadul, Roger Abounader

**Affiliations:** 1 Department of Microbiology, Immunology, and Cancer Biology, University of Virginia, Charlottesville, VA USA; 2 Department of Neurology, University of Virginia, Charlottesville, VA USA; 3 Department of Microbiology, Immunology, and Cancer Biology, Department of Neurology, University of Virginia, University of Virginia Cancer Center, Charlottesville, VA USA

**Keywords:** microRNAs, Brain, Metastasis, Biomarkers, Targets, Therapy

## Abstract

Brain metastasis (BM) is the most common type of brain tumor and frequently foreshadows disease progression and poor overall survival with patients having a median survival of 6 months. 70,000 new cases of BM are diagnosed each year in the United States (US) and the incidence rate for BM is increasing with improved detection. MicroRNAs (miRNAs) are small non-coding RNAs that serve as critical regulators of gene expression and can act as powerful oncogenes and tumor suppressors. MiRNAs have been heavily implicated in cancer and proposed as biomarkers or therapeutic targets or agents. In this review, we summarize an extensive body of scientific work investigating the role of microRNAs in BM. We discuss miRNA dysregulation, functions, targets, and mechanisms of action in BM and present the current standing of miRNAs as biomarkers and potential therapeutics for BM. We conclude with future directions of miRNA basic and clinical research in BM.

## Introduction

 Brain metastasis (BM) is the most common type of brain tumor with approximately 70,000 new cases diagnosed every year in the United States (U.S.) [1]. BM has an estimated incidence rate of 9–45% and has been increasing each year due to improved detection rates [2, 3]. As there are more effective treatments for primary cancers, the incidence of metastases to the central nervous system (CNS) is expected to increase. The most common types of cancers that metastasize to the brain are lung (16–36%), breast (5–30%), and melanoma (6–11%) [1, 4, 5]. Limited understanding of BM pathogenic mechanisms presents a major obstacle to finding better therapies. Although some crucial steps of the brain metastatic cascade are recognized, the full metastatic process remains unclear [6].

MicroRNAs (miRNAs) are small non-coding RNAs that modify cell function by regulating gene expression and protein translation. To date, there are over 75,000 scientific publications on miRNAs in cancer and that number continues to grow each year. miRNAs can be powerful oncogenes and tumor suppressors, disease biomarkers, and attractive therapeutic targets. They play a crucial role in cancer by regulating cellular processes such as proliferation, differentiation, apoptosis, survival, motility, and morphogenesis [7]. miRNAs have been implicated in cancer initiation, progression, and metastasis.

In this review, we review the current standing of miRNAs as it relates to brain metastasis. We also discuss future directions of miRNA research in BM from both basic science and clinical perspectives.

## Brain metastases

### General concepts

In the US, lung cancer, breast cancer, and melanoma are responsible for almost 80% of all BM with the number of BM cases reflecting the incidence of the primary cancers [8]. 25% of patients with the diagnosis of metastatic non-small cell lung cancer will have BM. In the case of melanoma, almost 70% of those with metastatic disease will have CNS involvement.

Historically, BM heralds disease progression and poor survival due to rapid neurological deterioration. It is a significant clinical problem and is associated with poor prognosis and high morbidity [9]. The contemporary prognosis and treatment of BM depends on various factors, including the number and location of the metastases, the presence of metastases in other organs, and the clinical functional status of the patient [8]. The median survival from BM diagnosis to death is on average less than 6 months and can be as short as 2.3 months despite advances in early detection and new therapeutic modalities [2, 10]. Treatment requires a multidisciplinary approach that includes surgery, radiotherapy, chemotherapy, targeted therapies (small molecules and immunotherapy), and palliative care. Treatment goals include: local control, providing the best level of quality of life, and prolonging survival. Furthermore, morbidity and mortality may also be dictated by the primary cancer.

### Pathobiology of brain metastases

Organotropism, the predisposition for particular primary tumors to spread to a specific organ such as the brain, is mostly linked to neoplastic cell intrinsic properties and the metastatic site microenvironment [11]. The metastatic cellular cross-talk is a complex process involving molecular mechanisms that modulate the microenvironment of the metastatic site, migration of primary tumor cells, and eventual infiltration of primary tumor cells into the final invasion site.

The following cellular processes promote BM: (1) formation of the pre-metastatic niche, (2) transendothelial migration across the blood-brain barrier (BBB), and (3) synergistic interaction of the cells and metabolites in the brain microenvironment [11]. In recent years, there has been growing evidence of a role for microRNAs in regulating BM.

## miRNAs

### General concepts

**Fig. 1:**
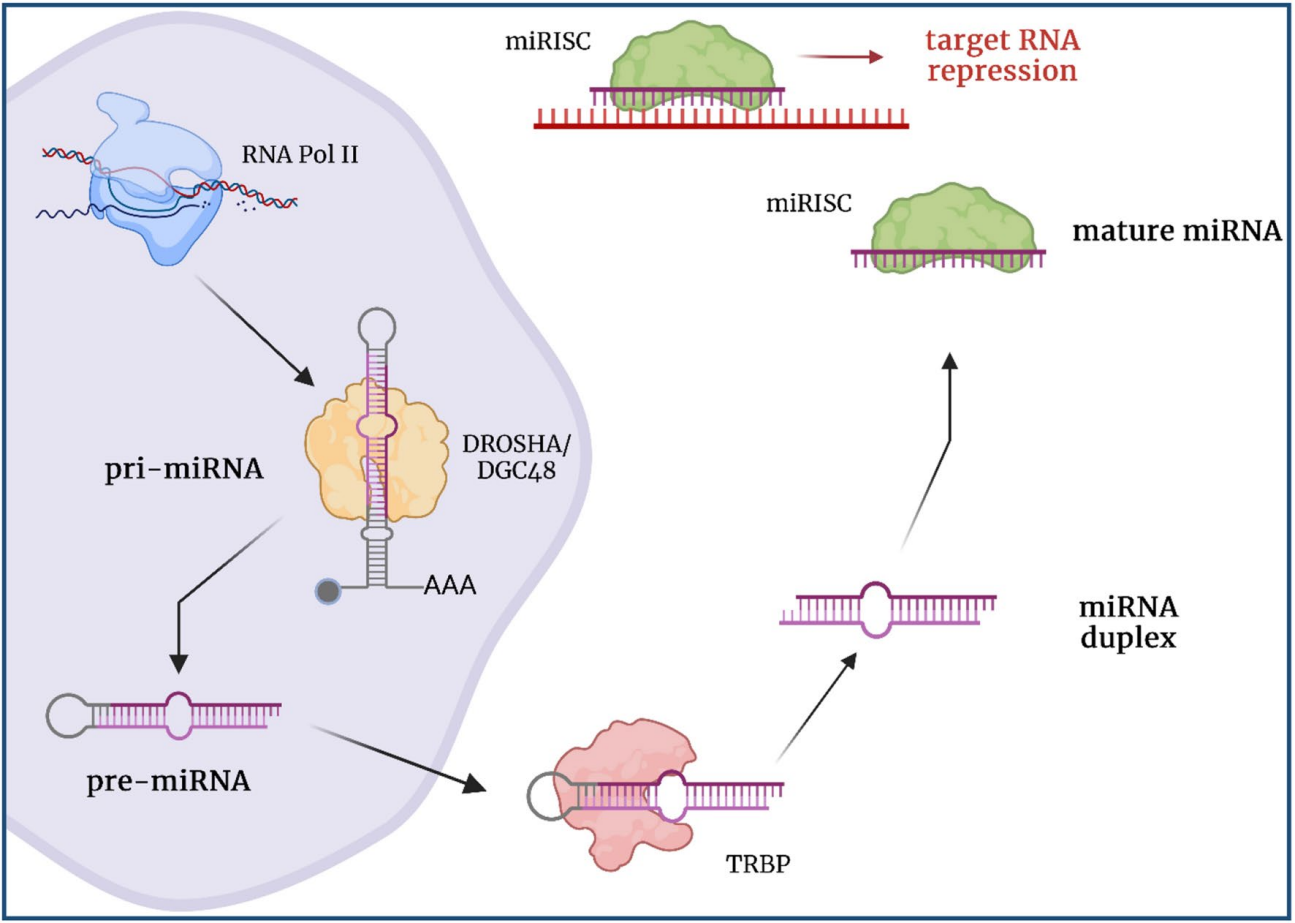
miRNA biogenesis and mechanism of action. Schematic illustrating miRNA biogenesis as the miRNA is transcribed and processed in the nucleus before being exported into the cytoplasm as a pre-miRNA. Once in the cytoplasm, the pre-miRNA is further processed into the mature miRNA that associates with the RISC complex to elicit target mRNA regulation. Created in BioRender.com

Non-coding RNAs (ncRNAs) represent a diverse class of functional molecules that comprise up to 98% of the human transcriptome [12]. One of the most well-characterized and significant types of ncRNAs are miRNAs. miRNAs are short, non-coding RNA transcripts that regulate gene expression and protein translation by binding to mRNA (Fig. 1). Since their discovery, miRNAs have been extensively implicated in a wide variety of human cancers [13]. Numerous studies have characterized miRNAs as critical oncogenes and tumor suppressors that regulate a variety of neoplastic processes including brain metastasis.

MiRNA genes are first transcribed as a primary miRNA (pri-miRNA). This pri-miRNA is cleaved into a precursor miRNA (pre-miRNA) before leaving the nucleus. Once in the cytoplasm, the pre-miRNA is cleaved into the mature miRNA. To exert their regulatory functions, mature miRNAs associate with the RNA-induced silencing complex (miRISC) which is composed of an Argonaute and other proteins. The miRNA wraps into the RISC complex, leaving the first 8 nucleotides of the 5’end free. These free nucleotides are termed the seed sequence and are critical for miRNA function. Complementarity between an RNA transcript and the miRNA seed sequence identifies miRNA targets and directs the miRISC complex. Once the miRNA seed sequence is bound to the RNA target, the miRISC complex blocks protein translation or initiates degradation of the RNA target (Fig. 1). Often, miRNAs bind to the 3’-UTR of mRNA targets to regulate their expression. However, miRNA binding to the 5’-UTR, coding region, and gene promoter region has also been observed [12]. miRNAs can target different types of cells including cancer cells and cells in the tumor microenvironment.

## miRNAs expression and functions in brain metastasis

### General concepts

A substantial body of scientific literature has focused on studying miRNA expression profiles and functions in primary tumors and BM. These studies have focused on lung cancers, breast cancers, and melanomas. Subsequent comparison of these miRNA expression profiles has revealed numerous miRNAs that are significantly dysregulated in BM. miRNA signatures outside the tumor and its microenvironment, particularly in blood, plasma, and cerebral spinal fluid (CSF) may be used as surrogate biomarkers. These biomarkers may identify patients with cancer and at high risk of developing BM, detect micrometastasis before it is detected on imaging studies, and characterize BM without requiring pathology and invasive brain surgery. Table 1 presents a summary of miRNAs that are dysregulated in BM of the most common types of cancers that metastasize to the brain.

**Table 1:** Oncogenic and tumor suppressive miRNAs in BM of the top 3 cancers that metastasize to the brain. Table 1 lists oncogenic miRNAs that are upregulated in BM (bold) and tumor suppressive miRNAs that are downregulated in BM (italic) from the three major primary tumors that metastasize to the brain

Primary tumor	miRNA	Reference(s)
Lung cancer	miR-9-5p	[19]
	miR-21-5p	[14]
	miR-184-5p	[24]
	miR-197-5p	[24]
	miR-328-5p	[20]
	miR-330-3p	[22, 28]
	miR-375-5p	[29]
	miR-378-5p	[15]
	miR-423-5p	[25]
	let-7a	[27]
	miR-145-5p	[17, 18, 21]
	miR-199a-5p	[19]
	miR-217-5p	[16]
	miR-596-3p	[23]
	miR-1207-5p	[30]
Breast cancer	miR-21-3p	[38]
	miR-92-1-5p	[31]
	miR-122-5p	[35]
	miR-181-1-3p	[31]
	miR-181c-5p	[33]
	miR-200 family	[38]
	miR-205-5p	[31]
	miR-503-5p	[34]
	miR-7-5p	[2]
	miR-126-5p	[37]
	miR-146a-5p	[2]
	miR-195-5p	[31]
	miR-196s-5p	[36]
	miR-199a-5p	[2]
	miR-211-5p	[2]
	miR-509-5p	[31]
	miR-802-5p	[41]
Melanoma	miR-518a-5p	[41]
	miR-527-5p	[41]
	miR-575-5p	[41]
	miR-622-5p	[41]
	miR-671-5p	[43]
	miR-4501-5p	[41]
	miR-4654-5p	[41]
	miR-4664-3p	[43]
	miR-4665-3p	[43]
	miR-4698-5p	[41]
	miR-5694-5p	[43]
	miR-6741-3p	[43]
	miR-6759-5p	[41]
	miR-6796-3p	[43]
	miR-8078-5p	[41]
	miR-34a-3p	[41]
	miR-224-5p	[39]
	miR-452-5p	[39]
	miR-542-3-5p	[40]
	miR-548f-4-5p	[41]
	miR-1270-5p	[41]
	miR-1290-5p	[41]
	miR-4278-5p	[41]
	miR-4528-5p	[41]

#### Lung cancer

Brain metastasis is a frequent complication associated with both small-cell and non-small-cell lung cancer (NSCLC). Many miRNAs in primary cancers have been correlated with the development of BM such as miR-21 in lung cancer patients [14]. Another oncogenic miRNA in lung cancer BM is miR-378 whose activity promotes higher expression of VEGF [15]. Downregulation of the following miRNAs is related to a higher incidence of BMs: miR-217, which is involved in the activation of the oncogenic protein p53, and miR-145, whose activity upregulates EGFR, thus promoting mitosis and cancer invasiveness [16–18]. Analysis of paired primary lung cancer and BM tissue biopsy identified upregulation of miR-423-5p, miR-197, miR-184, miR-9, miR-330-3p, miR-328 and downregulation of miR-199a-5p and miR-596-3p in BM [19–26].

The evidence for miRNAs as biomarkers in lung carcinoma BMs is scant. miR-let-7a is correlated with radiosensitivity of BMs, miR-330-3p influences the ability of lung cancer cells to metastasize, miR-375 is correlated with the incidence and prognosis of BMs, and miR-1207-5p inhibition promotes higher BBB permeability [27–30]. These miRNAs may represent predictive biomarkers.

#### Breast cancer

Fifteen to 25% of patients with metastatic breast cancer develop BM. Figueira, et al. reported that miR-802-5p and miR-195-5p were downregulated, while miR-92a-1-5p, miR-205-5p, and miR-181a-1-3p were upregulated in both tissue sample and plasma of breast cancer BM patients [31]. miRNA expression profiling of primary breast cancers and BM identified miR-199a-5p as downregulated in BM [32].

Downregulation of the following miRNAs was associated with the development of breast cancer BM: miR-7, miR-146a, miR-509, miR-211 [2]. In terms of BBB dysfunction, miR-181c promotes BBB destruction and miR-503 modifies the BBB microenvironment allowing cancer cells to pass [33, 34]. Additionally, miR-122 primes the BBB microenvironment as a premetastatic niche to support metastatic growth [35].

One study by Li et al. investigated the role of miRNAs in breast cancer cell migration and metastasis. The study found that the ratio of miR-196s to HOXC8 mRNA correlated with breast cancer cell migration and metastasis. Furthermore, the study demonstrated that knockdown of HOXC8 suppressed cell migration and metastasis, and ectopic expression of HOXC8 prevented the anti-tumoral effects of miR-196s on cell migration and metastasis [36]. These results suggest miR-196s as potential diagnostic biomarkers for BM.

miRNAs modulate the expression of cell adhesion molecules (CAMs) in cancer cells and endothelial cells, thereby facilitating the establishment of metastasis at distant metastatic sites. For example, miR-126 has been shown to suppress metastatic colonization by inhibiting pro-angiogenic genes and biomarkers of human metastasis [37]. Silencing of miR-126 in breast cancer cells leads to increased endothelial recruitment and metastatic brain colonization. Another miRNA, miR-21-3p, has been identified as a positive regulator of L1CAM expression, which is involved in the co-option of brain capillaries by cancer cells during metastasis [37]. Debeb et al. also noted that the miR-200 family was involved in the formation of breast BMs through higher expression of E-cadherin [38].

#### Melanoma

Melanoma is a malignant skin cancer that is notorious for metastasizing to the brain and causing intracranial bleeding and significant mortality. Downregulation of miR-224-5p, miR-452, and miR-542-3p promoted epithelial-to-mesenchymal transition (EMT) induction, thus increasing the invasiveness of melanoma cancer cells [39, 40].

miRNA expression profiling of primary melanomas with and without BM identified increased expression of miR-518a-5p, miR-527, miR-575, miR-622, miR-4501, miR-4654, miR-4698, miR-6759-5p, and miR-8078, and decreased expression of miR-150-5p, miR-34a-3p, miR-548f-4, miR-1270, miR-1290, miR-4278, and miR-4528 was noted [41, 42]. Bustos, et al. identified six miRNAs that were specifically increased in melanoma BMs compared to other types of BMs: miR-671-5p, miR-4664-3p, miR-4665-3p, miR-5694, miR-6741- 3p, and miR-6796-3p [43].

## miRNA targets and mechanisms of action in brain metastasis

### miRNAs promoting metastatic spread to the brain

miRNAs are important regulators of the tumor microenvironment as described in Table 2. Expression of miR-1258 in breast cancer BM cells inhibited the activity of heparanase (HPSE), a prometastatic enzyme that degrades components of the extracellular matrix (ECM) to increase cell motility, migration, and invasion. HPSE is a direct target of miR-1258; thus, miR-1258 is a tumor-suppressive miRNA in breast cancer BM that functions by upholding ECM integrity [44].

Additionally, miRNAs modulate BBB permeability and therefore are important regulators of tumor cell extravasation into the brain microenvironment. In NSCLC, miR-1207-5p targets EPB41L5. EPB41L5 is an oncogene that induces EMT, disrupts tight junctions, and increases BBB permeability. Thus, miR-1207-5p plays an important tumor suppressive role by maintaining BBB integrity and preventing metastatic spread to the brain [30]. miR-596-3p targets YAP1 in NSCLC and inhibits YAP1-induced MMP-2 activity which degrades tight junctions involved in BBB permeability [23]. Likewise, miR-509 targets TNFα and RhoC in breast cancer BM which inhibits RhoC-induced MMP-9 activity, disrupting tight junctions in the BBB [45]. In glioma vascular endothelial cells, miR-144 targets HSF2, miR-34c targets MAZ, miR-181d-5p targets SOX5, and miR-140 targets NFYA. All of these targets are crucial for promoting the expression of tight junction proteins such as ZO-1, occludin, and claudin-5 [46–49]. Lastly, miR-105 was shown to directly target ZO-1, an important tight junction protein, in breast cancer BM [50].

### miRNAs remodeling the brain microenvironment

**Fig. 2:**
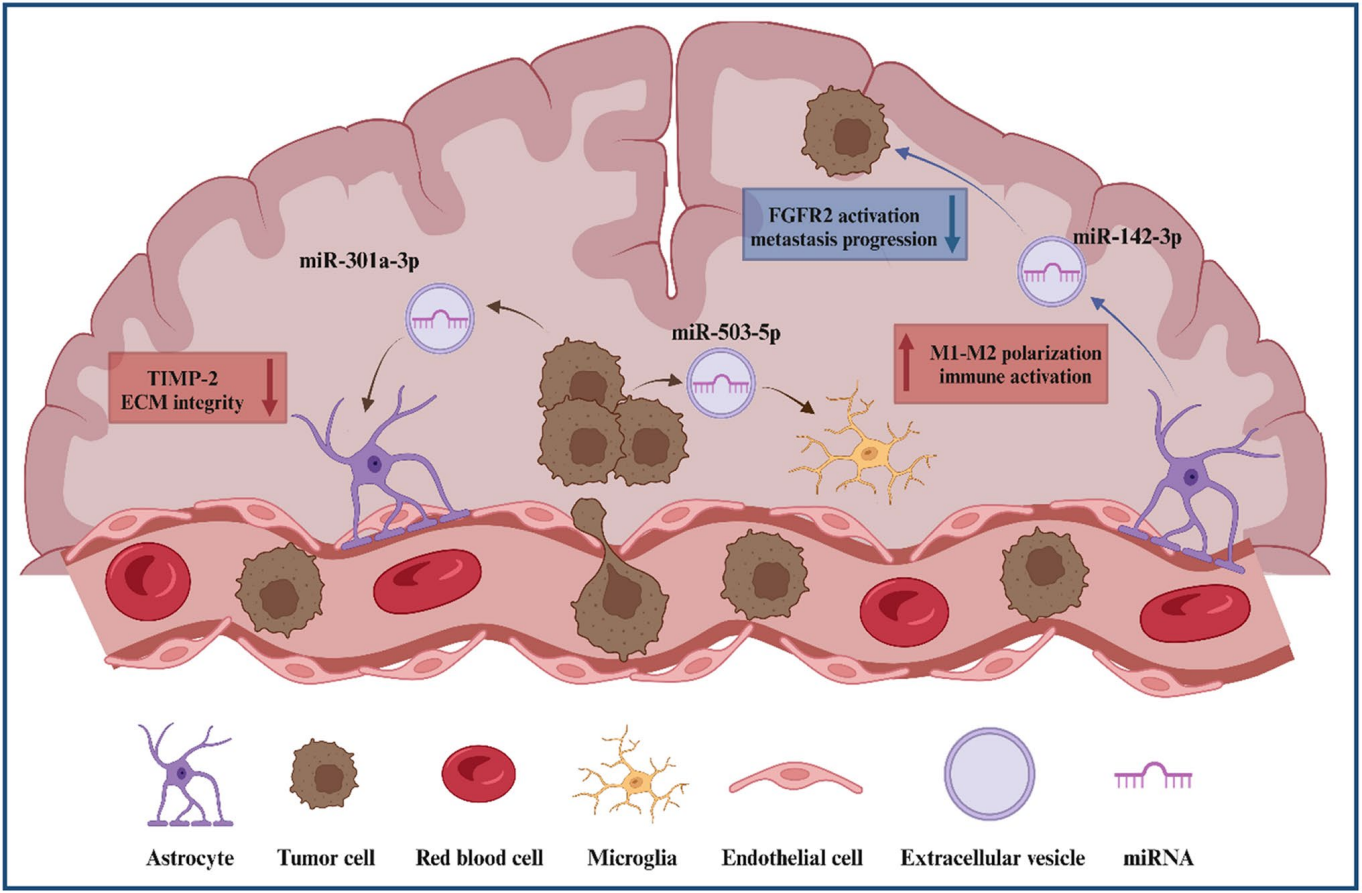
miRNA functions in the brain microenvironment. Figure 2 illustrates miRNA functions and mechanisms of action in the brain microenvironment. miR-142-3p is shown downregulating metastasis progression through FGFR2 inactivation, miR-503-5p is shown downregulating immune activation, and miR-301a-3p is shown down regulating ECM integrity. Red boxes and red arrows indicate oncogenic functions. Blue boxes and blue arrows indicate tumor suppressive functions. Created in BioRender.com

In addition to promoting metastatic spread to the brain, miRNAs remodel the brain microenvironment to foster BM growth. miRNAs significantly facilitate interactions between tumor cells and astrocytes, microglia, and endothelial cells to support BM as illustrated in Fig. 2.

Upregulated miR-345 then targets KISS1, a prominent tumor suppressor [53]. Thus, interactions between astrocytes and BM cells are mediated by miRNAs and significantly contribute to the remodeling of the brain microenvironment (See Table 2).

**Table 2:** miRNA targets and associated functional effects in brain metastasis

miRNA	Target(s)	Oncogene/tumor suppressor (miRNA)	miRNA source	Function	Reference(s)
miR-9-5p	CDH1	Tumor Suppressor	Microglia	Blocks MET and brain metastasis	[54]
miR-34c-5p	MAZ	Oncogene	Glioma endothelial cells	Promotes ECM degradation	[47]
miR-105-5p	ZO-1	Oncogene	Tumor cell derived EVs	Promotes ECM degradation	[50]
miR-122-5p	PKM	Oncogene	Tumor cell derived EVs	Modifies glucose utilization by pre-metastatic niche cells and reprograms systemic energy metabolism	[35]
miR-126-5p	VEGF, PI3K, MAPK, IGFBP2, PITPNC1, MERTK	Tumor Suppressor	Endothelial cells Tumor cells	Inhibits intravasation, metastatic endothelial recruitment, angiogenesis, and colonization	[37]
miR-140-5p	NFYA	Oncogene	Glioma endothelial cells	Promotes ECM degradation	[49]
miR-142-3p	TRPA1	Tumor Suppressor	Astrocytes	Inhibits FGFR2 activation and brain metastasis progression	[51]
miR-144-5p	HSF2	Oncogene	Glioma endothelial cells	Promotes ECM degradation	[46]
miR-145-5p	OCT-4, EGFR, MYC, TPD52	Tumor Suppressor	Tumor cells	Inhibits proliferation and migration, regulates EGFR signaling	[17, 18]
miR-181c-5p	PDPK1	Oncogene	Tumor cell derived EVs	Modulates actin dynamics and promotes BBB destruction	[33]
miR-181d-5p	SOX5	Oncogene	Glioma endothelial cells	Promotes ECM degradation	[48]
miR-196a1-5p	HOXC8	Tumor Suppressor	Unknown	Inhibits invasion, migration, and invasion	[36]
miR-196a2-5p	HOXC8	Tumor Suppressor	Unknown	Inhibits invasion, migration, and invasion	[36]
miR-196b-5p	HOXC8	Tumor Suppressor	Unknown	Inhibits invasion, migration, and invasion	[36]
miR-217-5p	Sirtuin 1	Tumor Suppressor	Tumor cells	Inhibits invasion, migration, and proliferation	[16]
miR-301a-3p	TIMP-2	Oncogene	Tumor cell derived EVs	Promotes ECM degradation	[52]
miR-330-3p	GRIA3	Oncogene	Tumor cells, Serum	Promotes cell proliferation, migration, invasion, EMT, angiogenesis, and G1/S phase transition Inhibited apoptosis	[28]
miR-345-5p	KISS1	Oncogene	Tumor cells	Promotes brain metastasis progression	[53]
miR-423-5p	MTSS1	Oncogene	Tumor cells	Increases colony formation, cell motility, migration, invasion	[25]
miR-509-5p	TNFa, RhoC	Tumor Suppressor	Tumor cells	Maintains ECM integrity	[45]
miR-596-3p	HPSE	Tumor Suppressor	Tumor cells	Maintains ECM integrity	[23]
miR-1207-5p	EPB41L5	Tumor Suppressor	Brain microvascular endothelial cells	Inhibits EMT, maintains tight junctions, inhibits BBB permeability	[30]
miR-1258-5p	YAP1	Tumor Suppressor	Tumor cells	Maintains ECM integrity	[44]

miRNAs also mediate BM by promoting interactions between tumor cells and microglia. The loss of the lncRNA XIST in breast cancer cells increases the secretion of exosomal miR-503 which triggers M1–M2 polarization of microglia. This polarization upregulates immune suppressive cytokines, ultimately leading to the suppression of T-cell proliferation in the brain microenvironment [34]. miRNAs are implicated in treatment-related remodeling of the brain microenvironment in BM. Irradiation of a BM NSCLC mouse model induced M1 microglial activation. Active M1 microglia increased secretion of miR-9, a miRNA that targets CDH1 and efficiently blocks mesenchymal to epithelial transition to reduce BM [54].

## Therapeutic role of miRNAs in brain metastasis

Brain metastasis is a complex and challenging condition to treat, and there is growing interest in exploring the role of miRNAs in its treatment. In theory, there are two general strategies for treating metastasis using miRNAs. The first is to decrease expression of oncogenic miRNAs using miRNA inhibitors, which are antisense oligonucleotides that block the pro-metastatic miRNAs. The second is to increase the expression of tumor suppressor miRNAs, which results in suppression of oncogenic pathways and cancer cell growth [3].

**Fig. 3:**
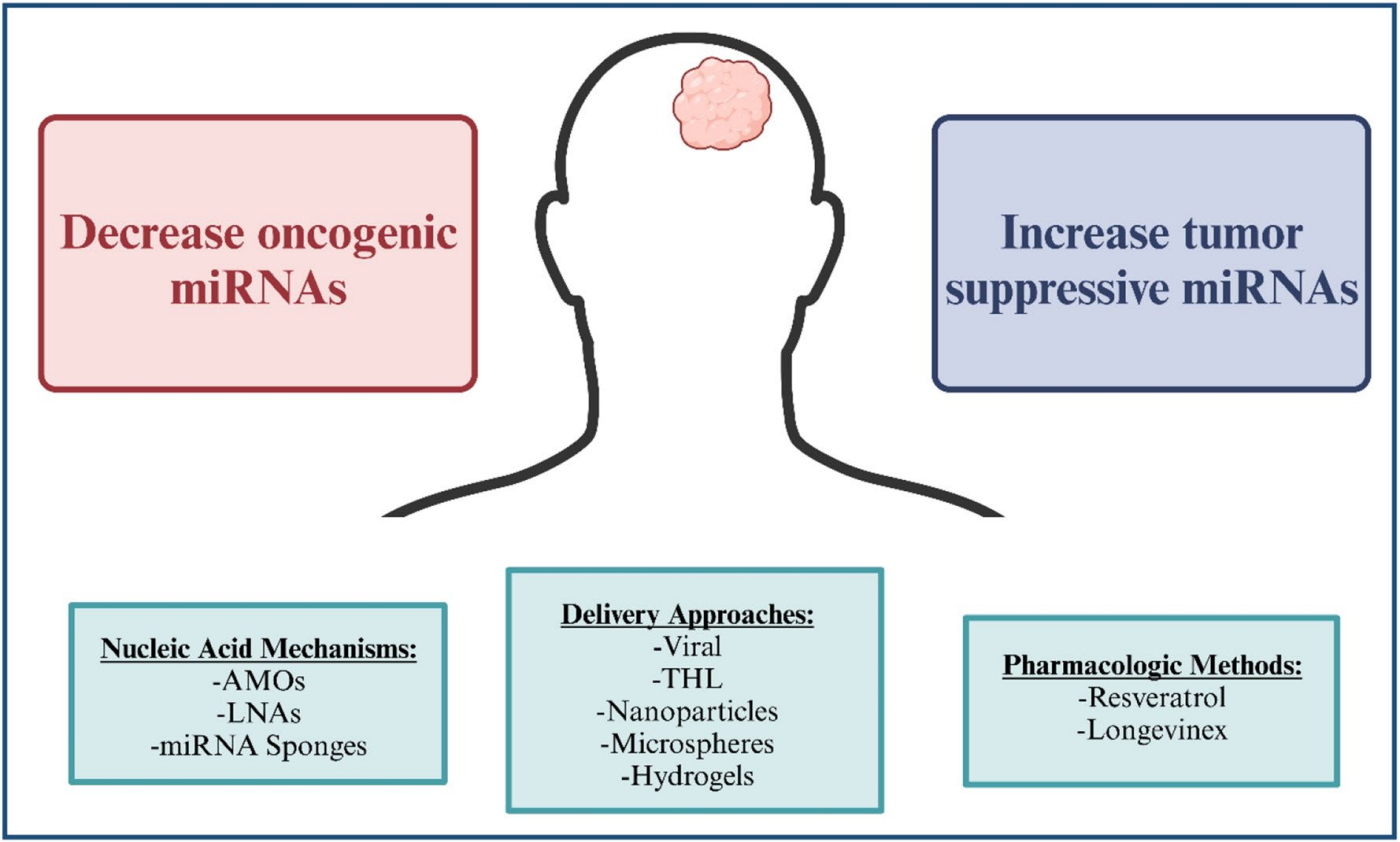
miRNAs therapeutics for brain metastasis. Schematic summarizing the therapeutic capacity of miRNAs for brain metastasis. This image lists the two main objectives of miRNA therapeutics and describes nucleic acid mechanisms, delivery approaches, and pharmacologic methodologies used to achieve these objectives. Created in BioRender.com

Synthetic anti-miRNA oligonucleotides (AMOs), locked nucleic acid (LNA)-anti-miRNAs, and miRNA sponges have been used for miRNA tehrapeutics [3, 55]. While these nucleic acid classes improve miRNA stability and regulation, they poorly penetrate the BBB. Viral and non-viral delivery methods have been used to address the challenges in miRNA therapeutic delivery (i.e. stability in blood and crossing the BBB). Using viruses to deliver anti-oncogenic miRNAs or specific tumor suppressor miRNAs shows potential for therapeutic use [56]. Additionally, non-viral delivery methods using liposomes in the form of neutral lipid emulsion through the Trojan Horse Liposome (THL) system, nanoparticles, microspheres, and hydrogels are advantageous as stable formulations [57, 58]. While non-viral delivery mechanisms have lower toxicity, viral delivery mechanisms have higher delivery efficiency [37]. The use of pharmacological agents like Resveratrol and Longevinex to alter miRNA levels has also been tested [59]. While these agents have reduced miRNA specificity, they offer an alternative miRNA targeting strategy compared to nucleic acid methodologies. These nucleic acid therapeutic mechanisms, delivery approaches, and pharmacologic methodologies are shown in Fig. 3.

A review by Jafri in 2017 identified miR-96, miR-182, miR-21, miR-181-3p, miR-503-3p, miR-135a/b, miR-155, and miR-520c as oncogenic miRNAs that may be targeted for inhibition. While miR-200, miR-655, miR-101, miR-205, miR-132, miR-21, miR-135b, miR-155, miR-182, miR10b, miR-148a, miR-215, miR-612, miR-191 and miR-192 have potential for miRNA replacement in anti-metastasis therapy [60]. Currently, only a few miRNAs have good evidence to support their use for therapy for metastasis from melanoma (miR-1258) and breast cancer (miR-33) [56]. Altogether, miRNA-based therapeutics that directly target BM are currently lacking.

## Future directions

While there is a significant amount of work depicting the mechanisms of action for many miRNAs in BM, there are numerous dysregulated miRNAs for which no functional understanding exists. Further research is needed to fully elucidate how miRNAs strengthen primary cancer cells for metastatic spread, prime the brain microenvironment for metastatic seeding, and promote BM growth. A comprehensive understanding of the mechanisms and pathways underlying pro-metastatic miRNA dysregulation in primary tumor cells may uncover novel therapeutic targets for preventative treatment.

In recent years, the research community has begun to understand the complex relationships that exist between tumor cells and resident cells of the brain. Cells such as astrocytes and neurons have been shown to communicate with tumor cells to promote or inhibit tumor growth. In this review, we discussed the few studies that have begun to investigate how this communication may occur within BM cells and the role of miRNAs in this communication. Further studies dedicated to revealing these mechanisms of communication in the brain microenvironment are crucial for understanding the roles of miRNAs and developing successful treatments for BM. Additionally, how miRNAs may be promoting cell state switching between the primary tumor environment and the brain microenvironment is a promising area of future research.

miRNAs as biomarkers for BM may be used for early detection of cancer progression. Prophylactic treatment of brain micrometastasis (i.e. absence of overt imaging evidence and non-symptomatic) usually with whole-brain radiation is controversial and may not be beneficial in BM due to the high morbidity related to radiation treatment effects. Validation of data on the classification of tissue origin of BMs through miRNAs will be beneficial in the clinical setting in cases of unknown primary tumors. The role of miRNAs in angiogenesis and EMT for prevention of BM, as well as their ability to regulate the tumor microenvironment to halt the progression and sensitize the cancer cells to chemotherapy are areas that need further investigation. Lastly, trends in miRNA expression during treatment can be potentially used as prognostic markers for predicting treatment response. Further translational research is needed to explore miRNAs as potential diagnostic markers and therapeutic agents or targets for patients with BM. Robust data regarding the type of sample (CSF, blood, or urine), standardized cut-off levels, and effective delivery routes are crucial for this translation.

## Conclusion

This review illustrates the crucial roles that miRNAs play in brain metastasis. We summarized the dysregulation of miRNAs in brain metastasis that originates from a wide variety of primary tumors and the potential use of miRNAs as biomarkers. We discussed how miRNAs can promote metastatic spread to the brain and remodel the brain microenvironment to support or inhibit brain metastasis progression. More research is needed before miRNAs can be exploited for BM therapy.

## Data Availability

This manuscript did not generate, analyze, or store any primary data.
